# Prevalence of Oral Fungal Infections Among Hemodialysis Patients and Their Association With Clinical and Demographic Factors: A Retrospective Study

**DOI:** 10.7759/cureus.107053

**Published:** 2026-04-14

**Authors:** Abedalelah M Al Momani, Loay Al Hammad, Nabeel Al Majali, Batool Al Zgoul, Abdalrahman Hamed, Alaa Khuluqi

**Affiliations:** 1 Oral and Maxillofacial Surgery, Prince Rashid Bin Al-Hassan Military Hospital, Royal Medical Services, Irbid, JOR; 2 Oral and Maxillofacial Surgery, Jordanian Royal Medical Services, Amman, JOR; 3 Orthodontics, Jordanian Royal Medical Services, Amman, JOR; 4 Periodontics, Jordanian Royal Medical Services, Amman, JOR; 5 Conservative Dentistry, Jordanian Royal Medical Services, Amman, JOR

**Keywords:** antifungal resistance, candida, fungal infections, hemodialysis, jordan, prophylaxis

## Abstract

Background: Patients on hemodialysis are more prone to having oral fungal infections due to weakened immunity, frequent medication use, and dry mouth. Understanding how clinical and lifestyle factors are associated with fungal presence may help in early detection and prevention. Clinically evident oral candidiasis is the most commonly observed fungal infection in hemodialysis patients, with diabetes and xerostomia as key associated risk factors, but available studies are mostly small, cross-sectional, and lack robust retrospective national data or systematic analysis of oral hygiene counseling practices.

Objective: We aimed to assess the prevalence of oral candidiasis in hemodialysis patients and explore their association with demographic and clinical features.

Methodology: We conducted a retrospective cross-sectional, single-center study at Prince Rashid Bin Al-Hassan Military Hospital between January 2024 and January 2025 on patients who were on regular hemodialysis. We collected basic demographic information from medical records and assessed the presence of oral fungal infection, along with its severity and whether the patients experienced pain or burning sensations. Hemodialysis-related variables, such as the number of dialysis sessions per week and duration on dialysis in months, were collected. We also gathered data on medication use, fasting and postprandial blood sugar, glycated hemoglobin, and duration of diabetes.

Results: Among 268 hemodialysis patients (161 males, 60%), the mean dialysis duration was 55 ± 34 months. Oral candidiasis was present in 147 (55%) patients: 39 (15%) had mild, 66 (25%) had moderate, and 42 (16%) had severe candidiasis. A total of 64 (24%) patients had positive potassium hydroxide/culture. Xerostomia occurred in 153 (57%), angular cheilitis in 83 (31%), and denture stomatitis in 86 (32%) patients. Lesions involved the tongue in 88 (33%), buccal mucosa in 67 (25%), palate in 64 (24%), and commissures in 54 (20%) patients. Compared with patients without candidiasis, those affected were more often current smokers (26% vs. 8.3%, p < 0.001), denture wearers (partial: 26% vs. 14%; complete: 14% vs. 7.6%, p = 0.006), and xerostomic (72% vs. 39%, p < 0.001); recent steroid exposure was higher (11% vs. 1.7%, p = 0.006). Diabetes (52% vs. 46%, p = 0.40), hypertension (78% vs. 81%, p = 0.80), immunosuppression (12% vs. 9.5%, p = 0.70), mouthwash use (48% vs. 44%, p = 0.70), dialysis sessions/week (p = 0.60), access type (p = 0.30), and dialysis duration (55 vs. 55 months, p = 0.80) did not differ. In multivariable analysis, independent predictors were current smoking (OR: 5.75, 95% CI: 2.13-17.4; p = 0.001), xerostomia (OR: 3.72, 95% CI: 1.89-7.53; p < 0.001), partial denture use (OR: 2.67, 95% CI: 1.10-6.87; p = 0.039), and recent steroid use (OR: 9.56, 95% CI: 1.45-192; p = 0.016). Diabetes (OR: 0.96; p > 0.90) and dialysis duration (OR: 1.00; p = 0.50) were not associated. Only 57 (22%) received antifungals, and 34 (13%) were treated at assessment, indicating undertreatment.

Conclusion: In hemodialysis patients, oral candidiasis is highly prevalent and independently associated with current smoking, denture use, xerostomia, and recent steroid therapy. High-risk patients should be identified at an early stage, and preventive measures should be focused on them. Timely antifungal therapy should be taken to reduce morbidity and enhance oral health outcomes among this vulnerable group.

## Introduction

Patients on hemodialysis often live with multiple health challenges that weaken their immune system and overall quality of life [1]. One of these issues is the development of oral fungal infections, especially oral candidiasis [2]. Oral candidiasis refers to infection of the mouth and throat by *Candida* yeast, most often *Candida albicans*. It typically presents as white patches or erythema with symptoms like soreness, burning, altered taste, or difficulty swallowing [3]. *Candida* colonization affects approximately 40-45% of those on hemodialysis, and is higher among denture wearers and those with dry mouth and low serum albumin [4].

These infections are uncomfortable to the patient, causing pain, burning, or difficulty eating, but can also indicate more complex systemic imbalances in these patients, including diabetes and poor salivary gland function [5]. Several studies have shown that individuals undergoing hemodialysis are more likely to experience oral candidiasis than the general population due to some factors such as chronic immunosuppression, dry mouth, and frequent use of broad-spectrum antibiotics or steroids [6]. Diabetes mellitus, which is common among dialysis patients, is associated with high blood sugar levels that feed the fungus and weaken the immune system [7]. Moreover, routine hemodialysis may disrupt the normal oral microbiome, creating an environment where opportunistic fungi such as *Candida albicans* can thrive [8]. Despite this, the oral health of patients on dialysis is frequently neglected both in clinical practice and research [9]. In addition to being a localized fungal infection, oral candidiasis in hemodialysis patients may reflect systemic immunological impairment and chronic inflammation. End-stage renal disease is among the conditions with a chronic inflammatory burden that are known to contribute to cardiovascular morbidity and mortality [2]. Many studies link oral candidiasis in hemodialysis patients to higher mortality, particularly from cardiovascular causes [2]. However, hemodialysis units may themselves harbor fungal organisms, they can support the growth of filamentous fungi and *Candida* biofilms, which resist common antifungals like micafungin and amphotericin B, but may also act as persistent reservoirs for reinfection via dialysis equipment [10].

We aim in this study to assess the prevalence of oral candidiasis in hemodialysis patients and explore their association with demographic and clinical features.

## Materials and methods

Study design

This retrospective cross-sectional study was conducted among hemodialysis patients at Prince Rashid Bin Al-Hassan Military Hospital, Irbid, Jordan, between January 2024 and January 2025. The study aimed to assess the prevalence of oral candidiasis and its association with demographic and clinical factors.

Data collection

A total of 268 patients undergoing regular hemodialysis were included in this study. Patients’ information was extracted from our institution’s electronic medical records, and data were then collected on an Excel sheet (Microsoft Corporation, Redmond, WA) for further analysis. Demographic variables such as age, gender, and smoking history, along with a battery of clinical variables, were collected; missing data were handled using a listwise deletion method.

Oral candidiasis was diagnosed using a standardized clinical protocol, which included inspection for characteristic white plaques, erythema, or soreness on the oral mucosa. When necessary, oral swabs were obtained for microscopic examination and fungal culture to confirm the diagnosis. All assessments were performed by trained clinicians familiar with oral fungal infections. Patients were then grouped according to the presence or absence of oral candidiasis and dialysis duration (<36 vs. >36 months), and comparative analyses were conducted to explore associations with demographic and clinical characteristics.

Patients with oral candidiasis were classified according to severity as follows: mild: localized lesions without symptoms; moderate: lesions with mild discomfort or burning; severe: extensive lesions causing significant pain or interfering with oral intake. Assessments were performed by trained clinicians using standardized criteria to ensure consistency.

Ethical considerations

The institutional review board at the Jordanian Royal Medical Services (JRMS) approved this study. The need for informed consent was waived due to the retrospective nature of the study. All data were deidentified prior to analysis.

Statistical methods

Data were analyzed using both descriptive and inferential statistical methods. Categorical variables such as sex, smoking status, denture use, comorbidities, and oral lesion characteristics were summarized as frequencies and percentages, while continuous variables like dialysis duration were presented as mean ± standard deviation. Group comparisons between patients with and without oral candidiasis (for comparative analysis, patients with uncertain clinical diagnosis were categorized within the non-candidiasis group), as well as by dialysis duration categories, were performed using Pearson’s chi-squared test for categorical variables and Welch’s two-sample t-test for continuous variables. Logistic regression models were applied to identify independent predictors of oral candidiasis, with odds ratios (OR) and 95% confidence intervals (CI) reported for each covariate. Additionally, ordinal logistic regression was used to evaluate factors associated with candidiasis severity, estimating the odds of progression from mild to severe disease. A p-value < 0.05 was considered statistically significant. All statistical analyses were done using R statistical software (version 4.3.0, R Foundation for Statistical Computing, Vienna, Austria).

## Results

In this study on the prevalence of oral candidiasis among hemodialysis patients, a total of 268 participants were analyzed. The demographic structure was geared toward males (161, 60%) more than females (107, 40%), with the minority declaring that they had a history of smoking, including 48 (18%) current smokers and 51 (19%) ex-smokers, whereas the majority were non-smokers (169, 63%). Denture wearing was low, with 182 (68%) denture-free, 56 (21%) wearing partial, and 30 (11%) wearing complete sets. More than half of the patients (153, 57%) reported experiencing xerostomia, and nearly half (124, 46%) of the patients described using mouthwash. Only 86 (32%) patients have ever been advised about oral hygiene. Regarding comorbidities, 133 (49%) patients were diabetic, and 212 (79%) were hypertensive. Other risk factors were immunosuppression in 30 (11%), recent antibiotic use in 54 (20%), and steroid use in 18 (6.8%). Nearly three-quarters of patients were dialyzed three times per week (212, 79%), with the majority having arteriovenous fistula (AVF) as their first choice of access type (153, 57%), and a smaller proportion of arteriovenous grafts (56, 21%) and central venous catheters (61, 23%) (Table 1).

**Table 1:** Baseline characteristics of the study population. Data are presented as n (%) or mean ± standard deviation. N = 268 hemodialysis patients.

Characteristic	Value
Male sex	161 (60.1%)
Female sex	107 (39.9%)
Current smoker	48 (17.9%)
Ex-smoker	51 (19.0%)
Never smoker	169 (63.1%)
Denture-free	182 (67.9%)
Partial denture	56 (20.9%)
Complete denture	30 (11.2%)
Xerostomia	153 (57.1%)
Mouthwash use	124 (46.3%)
Received oral hygiene counseling	86 (32.1%)
Diabetes mellitus	133 (49.6%)
Hypertension	212 (79.1%)
Immunosuppression	30 (11.2%)
Recent antibiotic use	54 (20.1%)
Recent steroid use	18 (6.7%)
Dialysis three times per week	212 (79.1%)
Arteriovenous fistula	153 (57.1%)
Arteriovenous graft	56 (20.9%)
Central venous catheter	61 (22.8%)
Dialysis duration (months)	55 ± 34

The frequency of oral candidiasis was high, affecting 147 (55%) of the patients; 113 (42%) had no infection, and eight (3%) remained uncertain. Severity among those infected varied: 39 (15%) had mild, 66 (25%) had moderate, and 42 (16%) had severe candidiasis. Laboratory diagnosis (potassium hydroxide (KOH) or culture) was done in 158 (59%) of patients, with 64 (24%) having a positive result. Antifungal treatment was used in 59 (22%) cases, mainly clotrimazole (32, 12%), nystatin (33, 12%), and fluconazole (13, 5%). Types of candidiasis were as follows: pseudomembranous (68, 46%), erythematous (84, 57%), and hyperplastic (15, 10%). Other orofacial findings were documented as follows: angular cheilitis (83, 31%), denture stomatitis (86, 32%), tongue infiltration (88, 33%), lesions on the palate (64, 24%), buccal mucosa lesions (67, 25%), and involvement of commissures (54, 20%). The average duration of dialysis was 55 months.

In this comparison between hemodialysis patients with and without oral candidiasis, several characteristics were significantly associated with the presence of infection. There was a significant difference in smoking (p < 0.001) (Table 2); the prevalence of current smoking was much higher in patients with candidiasis (38, 26.0%) than in those without candidiasis (10, 8.3%), and patients who never smoked were more likely to be in the non-candidiasis group (75% vs. 53%). Denture wearing was also statistically significant (p = 0.006); full (21, 14%) or partial (38, 26%) denture wearing was higher in patients with rather than without candidiasis (7.6% and 14%, respectively). Xerostomia (72% vs. 39%, p < 0.001) and recent steroid use (11% vs. 1.7%, p = 0.006) were significantly associated with candidiasis. Microbiologic confirmation effectively discriminated between the two groups; 64 (44%) patients with candidiasis had a positive KOH/culture finding in contrast with less than one (1%) patient without infection (p < 0.001). The clinical characteristics also differed significantly between the patients: angular cheilitis was more common among the candidiasis patients (38% vs. 21%, p = 0.005), and oral lesions were significantly more common in the tongue (60% vs. 0.8%), palate (43% vs. 0%), buccal mucosa (41% vs. 4.1%), and commissures (33% vs. 4.1%), all p < 0.001. These differences were mirrored in treatment patterns with antifungal treatment more prevalent among patients with candidiasis (32% vs. 8.8%, p < 0.001), and for specific agents including clotrimazole (20% vs. 1.7%), nystatin (18% vs. 5.0%), and fluconazole (8.8% vs. 0.8%), again with highly significant differences (p < 0.001).

**Table 2:** Comparison between patients with and without oral candidiasis. ^1 ^n/N (%); Mean (SD). ^2^ Pearson’s chi-squared test; Welch two sample t-test. AV: arteriovenous fistula; KOH: potassium hydroxide.

Characteristic	No Candidiasis/Uncertain (N = 121)^1^	Candidiasis (N = 147)^1^	Statistic value	P-value^2^
Gender			χ² = 2.43	0.12
Female	55 / 121 (45%)	52 / 147 (35%)	-	
Male	66 / 121 (55%)	95 / 147 (65%)	-	
Smoking status			χ² = 19.6	<0.001
Current	10 / 120 (8.3%)	38 / 145 (26%)	-	
Former	20 / 120 (17%)	30 / 145 (21%)	-	
Never	90 / 120 (75%)	77 / 145 (53%)	-	
Denture use			χ² = 10.3	0.006
Complete	9 / 119 (7.6%)	20 / 144 (14%)	-	
None	93 / 119 (78%)	86 / 144 (60%)	-	
Partial	17 / 119 (14%)	38 / 144 (26%)	-	
Xerostomia	47 / 120 (39%)	101 / 140 (72%)	χ² = 27.9	<0.001
Mouthwash use	52 / 118 (44%)	68 / 143 (48%)	χ² = 0.15	0.7
Oral hygiene counseling received	39 / 115 (34%)	41 / 138 (30%)	χ² = 0.28	0.6
Diabetes	51 / 112 (46%)	73 / 140 (52%)	χ² = 0.68	0.4
Hypertension	96 / 119 (81%)	113 / 144 (78%)	χ² = 0.06	0.8
Immunosuppression	11 / 116 (9.5%)	17 / 142 (12%)	χ² = 0.15	0.7
Recent antibiotics use in 30 days	16 / 115 (14%)	34 / 141 (24%)	χ² = 3.56	0.059
Recent steroids use in 30 days	2 / 118 (1.7%)	16 / 146 (11%)	χ² = 7.53	0.006
Sessions per week			χ² = 1.88	0.6
2	7 / 117 (6.0%)	6 / 146 (4.1%)	-	
3	94 / 117 (80%)	113 / 146 (77%)	-	
4	10 / 117 (8.5%)	20 / 146 (14%)	-	
Other	6 / 117 (5.1%)	7 / 146 (4.8%)	-	
Dialysis access type			χ² = 2.41	0.3
AV fistula	60 / 115 (52%)	85 / 141 (60%)	-	
AV graft	24 / 115 (21%)	29 / 141 (21%)	-	
Central catheter	31 / 115 (27%)	27 / 141 (19%)	-	
KOH or culture done	77 / 121 (64%)	82 / 147 (56%)	χ² = 1.64	0.2
KOH or culture result			χ² = 96.4	<0.001
Negative	76 / 121 (63%)	18 / 147 (12%)	-	
Not done	44 / 121 (36%)	65 / 147 (44%)	-	
Positive	1 / 121 (0.8%)	64 / 147 (44%)	-	
Antifungal use	10 / 114 (8.8%)	47 / 146 (32%)	χ² = 18.9	<0.001
Xerogenic meds	33 / 118 (28%)	41 / 142 (29%)	χ² = 0.01	>0.9
Treatment given today	0 / 121 (0%)	34 / 147 (23%)	χ² = 36.2	<0.001
Treatment agent			χ² = 38.5	<0.001
Clotrimazole	2 / 121 (1.7%)	29 / 147 (20%)	-	
Fluconazole	1 / 121 (0.8%)	13 / 147 (8.8%)	-	
Nystatin	6 / 121 (5.0%)	27 / 147 (18%)	-	
Other	1 / 121 (0.8%)	3 / 147 (2.0%)	-	
Subtype				
Pseudomembranous	52 / 121 (43%)	71 / 147 (48%)	χ² = 0.45	0.5
Erythematous	71 / 121 (59%)	82 / 147 (56%)	χ² = 0.15	0.7
Hyperplastic	15 / 121 (12%)	12 / 147 (8.2%)	χ² = 1.04	0.3
Angular cheilitis	26 / 121 (21%)	56 / 147 (38%)	χ² = 7.86	0.005
Denture stomatitis	31 / 121 (26%)	54 / 147 (37%)	χ² = 3.28	0.070
Site				
Tongue	1 / 121 (0.8%)	88 / 147 (60%)	χ² = 136.4	<0.001
Palate	0 / 121 (0%)	63 / 147 (43%)	χ² = 91.7	<0.001
Buccal mucosa	5 / 121 (4.1%)	61 / 147 (41%)	χ² = 68.2	<0.001
Commissures	5 / 121 (4.1%)	48 / 147 (33%)	χ² = 52.9	<0.001
Dialysis duration (months)	55 (32)	55 (36)	t = 0.25	0.8

Other factors, however, had no differences among the groups. The sex ratio did not differ by the presence of candidiasis compared with non-candidiasis cases (p = 0.12), with slightly more males in the candidiasis group (65% vs. 55%). There were no differences in mouthwash use (48% vs. 44%, p = 0.7), receipt of oral hygiene counseling (30% vs. 34%, p = 0.6), diabetes diagnosis (52% vs. 46%, p = 0.4), hypertension (78% vs. 81%, p = 0.8), and immunosuppression (12% vs. 9.5%, p = 0.7). Dialysis-related parameters such as the weekly number of dialysis sessions (p = 0.6), the type of access for dialysis (p = 0.3), and the duration of dialysis (55 months in both groups, p = 0.8) were also similar. Xerogenic medications did not differ (29% vs. 28%, p > 0.9). Subtypes of candidiasis (pseudomembranous, erythematous, and hyperplastic) were not significantly different between groups (all: p > 0.3), and denture stomatitis was only borderline associated (37% vs. 26%, p = 0.07).

When comparing short dialysis duration (<36 months) and long dialysis duration (≥36 months) among hemodialysis patients, the only significant variable between the two hemodialysis groups was dialysis duration: 26 ± 6 months for the short-duration group vs. 70 ± 32 months for the long-duration group (p < 0.001). None of the other demographic, clinical, and oral health-related factors were significantly associated with dialysis duration. In particular, sex, smoking, use of dentures, xerostomia, use of mouthwash, receiving oral hygiene instruction, diabetes, hypertension, immunosuppression, recent antibiotic or steroid use, dialysis sessions per week, and vascular access were similar between the groups (all p > 0.2, Table 3). Similarly, oral findings, KOH/culture results, use of current antifungals, xerogenic medication use, treatment provided, subtypes of candidiasis, angular cheilitis, denture stomatitis, and distribution of lesions (tongue, palate, buccal mucosa, commissures) were not significantly different.

**Table 3:** Comparison by dialysis duration (<36 vs. ≥36 months). ^1 ^n/N (%); Mean (SD). ^2 ^Pearson’s chi-squared test; Welch two sample t-test. AV: arteriovenous; KOH: potassium hydroxide.

Characteristic	Low (<36 m) (N = 91)^1^	High (≥36 m) (N = 177)^1^	Statistic value	P-value^2^
Gender			χ² = 1.07	0.3
Female	32 / 91 (35%)	75 / 177 (42%)	-	
Male	59 / 91 (65%)	102 / 177 (58%)	-	
Smoking status			χ² = 2.41	0.3
Current	19 / 89 (21%)	29 / 176 (16%)	-	
Former	20 / 89 (22%)	30 / 176 (17%)	-	
Never	50 / 89 (56%)	117 / 176 (66%)	-	
Denture use			χ² = 0.71	0.7
Complete	11 / 88 (13%)	18 / 175 (10%)	-	
None	61 / 88 (69%)	118 / 175 (67%)	-	
Partial	16 / 88 (18%)	39 / 175 (22%)	-	
Xerostomia	49 / 86 (57%)	99 / 174 (57%)	χ² <0.01	>0.9
Mouthwash use	38 / 88 (43%)	82 / 173 (47%)	χ² = 0.28	0.6
Oral hygiene counseling received	30 / 88 (34%)	50 / 165 (30%)	χ² = 0.27	0.6
Diabetes	39 / 84 (46%)	85 / 168 (51%)	χ² = 0.27	0.6
Hypertension	73 / 90 (81%)	136 / 173 (79%)	χ² = 0.06	0.8
Immunosuppression	10 / 86 (12%)	18 / 172 (10%)	χ² <0.01	>0.9
Recent antibiotics use in 30 days	21 / 87 (24%)	29 / 169 (17%)	χ² = 1.64	0.2
Recent steroids use in 30 days	7 / 89 (7.9%)	11 / 175 (6.3%)	χ² = 0.06	0.8
Sessions per week			χ² = 2.31	0.5
2	2 / 89 (2.2%)	11 / 174 (6.3%)	-	
3	72 / 89 (81%)	135 / 174 (78%)	-	
4	10 / 89 (11%)	20 / 174 (11%)	-	
Other	5 / 89 (5.6%)	8 / 174 (4.6%)	-	
Dialysis access type			χ² = 0.45	0.8
AV fistula	50 / 86 (58%)	95 / 170 (56%)	-	
AV graft	16 / 86 (19%)	37 / 170 (22%)	-	
Central catheter	20 / 86 (23%)	38 / 170 (22%)	-	
KOH or culture done	61 / 91 (67%)	98 / 177 (55%)	χ² = 2.95	0.087
KOH or culture result			χ² = 3.22	0.2
Negative	35 / 91 (38%)	59 / 177 (33%)	-	
Not done	30 / 91 (33%)	79 / 177 (45%)	-	
Positive	26 / 91 (29%)	39 / 177 (22%)	-	
Antifungal use	24 / 90 (27%)	33 / 170 (19%)	χ² = 1.64	0.2
Xerogenic meds	24 / 91 (26%)	50 / 169 (30%)	χ² = 0.15	0.7
Treatment given today	12 / 91 (13%)	22 / 177 (12%)	χ² <0.01	>0.9
Treatment agent			χ² = 4.62	0.2
Clotrimazole	8 / 91 (8.8%)	23 / 177 (13%)	-	
Fluconazole	7 / 91 (7.7%)	7 / 177 (4.0%)	-	
Nystatin	16 / 91 (18%)	17 / 177 (9.6%)	-	
Other	2 / 91 (2.2%)	2 / 177 (1.1%)	-	
Subtype				
Pseudomembranous	37 / 91 (41%)	86 / 177 (49%)	χ² = 1.07	0.3
Erythematous	52 / 91 (57%)	101 / 177 (57%)	χ² <0.01	>0.9
Hyperplastic	13 / 91 (14%)	14 / 177 (7.9%)	χ² = 1.64	0.2
Angular cheilitis	21 / 91 (23%)	61 / 177 (34%)	χ² = 3.15	0.076
Denture stomatitis	28 / 91 (31%)	57 / 177 (32%)	χ² <0.01	>0.9
Site				
Tongue	32 / 91 (35%)	57 / 177 (32%)	χ² = 0.15	0.7
Palate	22 / 91 (24%)	41 / 177 (23%)	χ² <0.01	>0.9
Buccal mucosa	18 / 91 (20%)	48 / 177 (27%)	χ² = 1.64	0.2
Commissures	16 / 91 (18%)	37 / 177 (21%)	χ² = 0.28	0.6
Dialysis duration (months)	26 (6)	70 (32)	t = −13.8	<0.001

Several variables were significant predictors of oral candidiasis among hemodialysis patients as determined by multivariable logistic regression. Current smoking had a strong association with infection (OR = 5.75, 95% CI: 2.13-17.4, p = 0.001, Table 4), while former smoking had a weaker, non-significant trend (OR = 1.95, 95% CI: 0.80-4.92). Denture wear was also a contributory factor (p = 0.039), and partial denture wear was associated with the highest odds (OR = 2.67, 95% CI: 1.10-6.87). There was a further but non-significant increase in the case of complete denture wear (OR = 2.96, 95% CI: 0.79-13.1). Xerostomia became a highly significant factor, with those who reported it being nearly four times more likely to have candidiasis (OR = 3.72, 95% CI: 1.89-7.53, p < 0.001). Recent steroid therapy was also an important risk factor (OR = 9.56, 95% CI: 1.45-192, p = 0.016). In contrast, other variables, including diabetes (OR = 0.96, p > 0.9), recent antibiotics (OR = 1.96, p = 0.2), immunosuppression (OR = 0.90, p = 0.8), mouthwash use (OR = 1.16, p = 0.7), oral hygiene counseling (OR = 0.56, p = 0.12), dialysis access type (p = 0.2), and duration of dialysis (OR = 1.00, p = 0.5) were not significantly associated with candidiasis.

**Table 4:** Logistic regression for predictors of oral candidiasis. OR: odds ratio, CI: confidence interval; AV: arteriovenous.

Characteristic		OR	95% CI		Wald χ²	P-value
			Lower	Upper		
Smoking status					13.8	0.001
Never		Reference				
Former		1.95	0.80	4.92	-	
Current		5.75	2.13	17.4		
Denture use					6.47	0.039
None		Reference				
Partial		2.67	1.10	6.87	-	
Complete		2.96	0.79	13.1		
Xerostomia					15.9	<0.001
No		Reference				
Yes		3.72	1.89	7.53	-	
Diabetes					<0.01	>0.9
No		Reference				
Yes		0.96	0.48	1.91	-	
Recent antibiotics use in 30 days					1.64	0.2
No		Reference				
Yes		1.96	0.75	5.38	-	
Recent steroids use in 30 days					5.79	0.016
No		Reference				
Yes		9.56	1.45	192	-	
Immunosuppression					0.06	0.8
No		Reference				
Yes		0.90	0.33	2.48	-	
Mouthwash use					0.15	0.7
No		Reference				
Yes		1.16	0.59	2.28	-	
Oral hygiene counseling received					2.41	0.12
No		Reference				
Yes		0.56	0.26	1.17	-	
Dialysis access type					3.22	0.2
AV fistula		Reference				
AV graft		0.76	0.32	1.77	-	
Central catheter		0.48	0.20	1.12		
Dialysis duration (months)		1.00	0.99	1.01	0.45	0.5

## Discussion

In this study, we observed a high prevalence of oral candidiasis (147, 55%) among hemodialysis patients, which aligns with prior reports suggesting that individuals with end-stage renal disease (ESRD) are particularly susceptible to oral fungal infections due to immunosuppression, xerostomia, and frequent exposure to medications [4,11,12]. The predominance of moderate to severe disease in nearly 40% of affected patients underscores the clinical significance of this condition and highlights the need for routine oral assessment in this population [13,14].

Our analysis identified recent steroid use as the strongest independent predictor of oral candidiasis (OR: 9.56), consistent with previous studies reporting that corticosteroids compromise local immunity, which may facilitate *Candida* overgrowth [12,14]. Similarly, smoking and xerostomia were significantly associated with candidiasis in univariate analyses, supporting prior findings that impaired salivary flow and mucosal microenvironment changes predispose patients to fungal colonization [12]. While diabetes mellitus was not independently predictive in our multivariate model, it remains a clinically relevant factor given its established association with oral fungal infections [15,16].

Denture use, particularly partial dentures, was also linked to candidiasis, while complete denture use showed a non-significant trend, likely due to denture-induced microtrauma and biofilm formation, echoing results from previous studies in both dialysis and general populations [15,17,18]. The involvement of multiple oral sites, including the tongue, palate, buccal mucosa, and commissures, mirrors the distribution patterns reported in the literature, with pseudomembranous and erythematous subtypes being most common [11,13]. Interestingly, dialysis-related factors such as session frequency, access type, and duration were largely not associated with candidiasis in our cohort. This suggests that local oral factors and medication exposure play a more prominent role than the dialysis procedure itself, a finding that aligns with some prior studies but contrasts with reports linking longer dialysis duration to increased infection risk [19].

Despite the high prevalence of candidiasis, only 59 (22%) patients were on antifungal therapy, and treatment on the day of assessment was provided to just 13%. Clotrimazole and nystatin were the most frequently used agents, reflecting standard clinical practice [15,20]. This apparent under-treatment highlights the need for enhanced screening and timely management, especially among high-risk patients such as those receiving steroids or with xerostomia.

Our study also emphasizes the importance of oral hygiene counseling, yet only 86 (32%) of patients reported receiving guidance, suggesting a gap in preventive care. Targeted interventions, including education, regular oral examinations, and prophylactic antifungal strategies for high-risk patients, may help reduce the burden of oral candidiasis in this population [15].

Limitations of our study include its single-center, retrospective, cross-sectional design, which may limit generalizability and preclude causal inference. Some variables had missing data, and we did not perform longitudinal follow-up to assess recurrence or treatment response. Future studies should incorporate larger, multicenter cohorts and evaluate the effectiveness of preventive and therapeutic interventions for oral candidiasis in dialysis patients.

## Conclusions

Oral candidiasis is highly prevalent among hemodialysis patients, with recent steroid use, smoking, xerostomia, and denture use identified as key associated factors. Enhanced screening, patient education, and timely antifungal treatment are essential to mitigate morbidity and improve oral health outcomes in this vulnerable population.
